# Anti-Tyro3 IgG Associates with Disease Activity and Reduces Efferocytosis of Macrophages in New-Onset Systemic Lupus Erythematosus

**DOI:** 10.1155/2020/2180708

**Published:** 2020-11-10

**Authors:** Zhuochao Zhou, Aining Xu, Jialin Teng, Fan Wang, Yun Tan, Honglei Liu, Xiaobing Cheng, Yutong Su, Hui Shi, Qiongyi Hu, Huihui Chi, Jian Li, Jiaqi Hou, Yue Sun, Chengde Yang, Junna Ye

**Affiliations:** ^1^Department of Rheumatology and Immunology, Ruijin Hospital, Shanghai Jiao Tong University School of Medicine, Shanghai, China; ^2^State Key Laboratory of Medical Genomics, Shanghai Institute of Hematology, Ruijin Hospital, Shanghai Jiao Tong University School of Medicine, Shanghai, China; ^3^Clinical Research Center, Ruijin Hospital, Shanghai Jiao Tong University School of Medicine, Shanghai, China; ^4^Department of Rheumatology and Immunology, Yueyang Hospital of Integrated Traditional Chinese and Western Medicine, Shanghai University of Traditional Chinese Medicine, Shanghai, China

## Abstract

**Background:**

Systemic lupus erythematosus (SLE) is a disease characterized by the production of a large number of autoantibodies. Defected phagocytosis of macrophage plays an important role in innate immunity in the pathogenesis of SLE. Tyro3 is a receptor responsible for the recognition of apoptotic cells during efferocytosis by macrophages. To investigate the role of Tyro3 receptor in macrophages' efferocytosis of apoptotic cells in SLE, we aimed to reveal the clinical relevance and impact of Tyro3 autoantibody on SLE.

**Methods:**

The serum levels of IgG-type autoantibody against Tyro3 receptor were detected in new-onset, treatment-naïve SLE patients (*n* = 70), rheumatoid arthritis (RA) (*n* = 24), primary Sjögren's Syndrome (pSS) (*n* = 21), and healthy controls (HCs) (*n* = 70) using enzyme-linked immunosorbent assay (ELISA). The effects of purified Tyro3 autoantibody from SLE patients on the efferocytosis of human monocyte-derived macrophages were measured by flow cytometry and immunofluorescence.

**Results:**

The serum levels of IgG-type autoantibody against Tyro3 receptor were significantly elevated in patients with SLE compared to RA, pSS, and HCs (all *p* < 0.0001). The levels of anti-Tyro3 IgG were positively associated with the SLE disease activity index (SLEDAI) score (*r* = 0.254, *p* = 0.034), erythrocyte sedimentation rate (ESR) (*r* = 0.430, *p* < 0.001), C-reactive protein (CRP) (*r* = 0.246, *p* = 0.049), and immunoglobulin G (IgG) (*r* = 0.408, *p* = 0.001) and negatively associated with haemoglobin (Hb) (*r* = −0.294, *p* = 0.014). ROC curves illustrated that the anti-Tyro3 antibody could differentiate patients with SLE from HCs. Furthermore, flow cytometry and immunofluorescence demonstrated that purified anti-Tyro3 IgG inhibited the efferocytosis of macrophages (*p* = 0.004 and 0.044, respectively) compared with unconjugated human IgG.

**Conclusions:**

These observations indicated that autoantibody against Tyro3 was associated with disease activity and could impair efferocytosis of macrophages. It might be a potential novel disease biomarker and might be involved in the pathogenesis of SLE.

## 1. Introduction

Systemic lupus erythematosus (SLE), which is characterized by the production of a large number of autoantigens and autoantibodies [1, 2], is a chronic autoimmune disease that affects almost all organs. A large number of studies have shown that functional defect of mononuclear macrophages, especially phagocytosis, plays an important role in innate immunity in the pathogenesis of SLE [3]. Numerous lines of evidence have revealed that the overproduction of autoantibodies is attributed to the impaired efferocytosis of apoptotic cells and debris [1, 4, 5]. Our group previously reported that autoantibodies against type A scavenger receptors, which was a type of receptors related to the efferocytosis of macrophages, could reduce its ability to clear apoptotic cells in lupus patients [6]. However, the mechanism underlying the impairment of efferocytosis in SLE is not fully understood.

Macrophages belong to the largest subset of immune cells that are principally responsible for efferocytosis [2, 7]. Generally, efferocytosis in macrophages is initiated by the recognition of apoptotic cells via receptors on the cell surfaces of macrophages, which then trigger the phagocytic process [8, 9]. A 3-member transmembrane kinase receptor family named Tyro3/Axl/Mertk (TAM) is responsible for the recognition of apoptotic cells during efferocytosis by macrophages [10]. TAM family receptors include the Tyro3, Axl, and Mertk receptor tyrosine kinases and can be activated by interaction with the “eat-me” phosphatidylserine on the surfaces of apoptotic cells, which rely on the assistance of the ligands growth arrest-specific 6 (GAS6) and protein S [11, 12]. Emerging evidence has revealed that a deficiency in TAM receptors results in the accumulation of apoptotic cells [4, 13]. For instance, Mertk knockdown or knockout mice showed the delayed clearance of infused apoptotic cells [14]. Not only Mertk but also Axl and Tyro3 function in the phagocytosis of apoptotic cells. Axl^−/−^ and Tyro3^−/−^ macrophages had ∼40–50% reduction in their abilities to phagocytose apoptotic thymocytes [15]. Furthermore, triple knockout Tyro3/Axl/Mertk mice developed SLE-like autoimmunity and overproduced autoantibodies, such as anti-double-stranded DNA (dsDNA) [16], indicating that the dysfunction of the TAM receptors on macrophages contributed to the development of SLE. Among TAM receptors, Tyro3 is the least characterized member. In the hematopoietic system, Tyro3 receptor is expressed on dendritic cells, natural killer cells, monocytes and macrophages, platelets and megakaryocytes, and osteoclasts [17]. It was reported that primary peritoneal macrophages isolated from Tyro3^−/−^ mice had decreased ability to phagocytose apoptotic cells compared to macrophages from wild-type mice [17]. Clinical studies revealed that the levels of the soluble forms of Tyro3 (sTyro3) and Mertk receptors were increased in plasma from SLE patients [18]. Both levels of soluble forms of Mertk (sMertk) and sTyro3 were correlated with SLEDAI, and levels of sTyro3 were higher in patients with higher SLEDAI [19]. It might be responsible for the defective function of efferocytosis in the disease [20]. However, whether Tyro3 receptor could serve as an autoantigen to further affect the function of macrophages has remained unexplored. Here, we systematically investigated the profiles and clinical relevance of the Tyro3 autoantibody in new-onset and treatment-naïve SLE patients and further explored the implications of this about the efferocytosis by macrophages.

## 2. Methods

### 2.1. Patients and Healthy Controls

The study included consecutive 70 new-onset and treatment-naïve patients with SLE (including 60 females and 10 males) who met the 1997 American College of Rheumatology (ACR) classification criteria for the diagnosis of SLE confirmed by two qualified rheumatologists (Jialin Teng and Chengde Yang) [21]. Demographic data, clinical characteristics, and laboratory findings such as anti-dsDNA IgG levels, erythrocyte sedimentation rate (ESR), white blood cell counts in blood (WBC), haemoglobin (Hb), platelets (PLT), C-reactive protein (CRP), immunoglobulin G (IgG), complement 3 (C3), and complement 4 (C4) of SLE patients were collected. In addition, 24 rheumatoid arthritis (RA) and 21 primary Sjögren's Syndrome (pSS) were enrolled, and samples from 70 sex- and age-matched healthy donors with neither autoimmune nor infectious diseases were collected as healthy controls (HCs) (including 58 females and 12 males). All of the sera samples were stored at -80°C until use. Disease activity was measured using the SLEDAI score [22]. The study was performed in accordance with the Declaration of Helsinki and the Principles of Good Clinical Practice. Biological samples were obtained under a protocol approved by the Institutional Research Ethics Committee of Ruijin Hospital (ID: 2016-62), Shanghai, China. We have obtained the written consent from recruited patients and HCs.

### 2.2. Detection of Anti-Tyro3 Autoantibody

Recombinant human Tyro3 protein (Abnova, Taiwan) was prepared by a wheat germ expression system. The protein was fused with a GST-tag at N-terminal and purified by glutathione sepharose 4 fast flow. Antibody against human Tyro3 receptor in the sera of SLE patients and HCs was determined by an enzyme-linked immunosorbent assay (ELISA) at 450 nm. Ninety-six-well high-binding plates (Corning, New York, USA) were coated with recombinant human Tyro3 protein overnight at 4°C. The antigen-coated wells were washed three times with PBST buffer (PBS plus 0.05% Tween-20) and blocked with PBST buffer containing 5% bovine serum albumin (BSA) for 2 h at 37°C. The human Tyro3/Dtk antibody (R&D Systems, USA) was used as a positive control. The blocking buffer was removed, and the plates were washed as described above before the addition of 100 *μ*l of serum sample (1 : 100 diluted in 1% BSA). The human sera were incubated for 2 h at room temperature followed by incubation with HRP-conjugated goat anti-human IgG (Abcam, Cambridge, UK) for another 1 h at room temperature. Then, the plates were washed, and 100 *μ*l of tetramethylbenzidine substrate solution was added. It was then stopped by the addition of 50 *μ*l of 0.5 M H_2_SO_4_. The absorbance was measured at a wavelength of 450 nm in a microplate reader (Bio-Rad Laboratories, Richmond, USA).

### 2.3. IgG Purification

Total IgG was isolated from the serum of new-onset SLE patients by a thiophilic adsorbent reagent (Pierce, Thermo Scientific, Rockford, USA) according to the manufacturer's instructions and concentrated in a centrifuge tube (Amicon, Millipore, Eschborn, Germany).

### 2.4. Purification of Anti-Tyro3 IgG

The purification of the specific antibody was performed using AminoLink Plus Coupling Resin according to the manufacturers' instructions (MicroLink, Thermo Scientific, USA). We purchased recombinant human Tyro3 protein (Abnova, Taiwan) and coupled the protein to the resin. Then, we used the purified total IgG from SLE patients to form the resin-bound complex and incubated them with gentle end-over-end mixing. The eluted antibody was neutralized with 1 M Tris (pH = 9.0). After sterile filtration, the autoantibody was stored as small aliquots at -80°C.

### 2.5. Preparation of Macrophages

Peripheral blood mononuclear monocytes (PBMCs) were isolated from the blood of healthy volunteers using Ficoll density gradient centrifugation (GE Healthcare, Madison, USA). The CD14+ monocytes were isolated by positive selection using CD14 microbeads (Miltenyi Biotec, Auburn, USA) according to the manufacturer's instructions. The selected cells were cultured in RPMI 1640 medium containing 10% fetal calf serum (FCS; Gibco, NY, USA), 100 units/ml penicillin, and 100 *μ*g/ml streptomycin that was supplemented with 100 ng/ml of macrophage colony-stimulating factor (M-CSF) (R&D Systems, Minneapolis, USA) in a humidified 5% CO_2_ incubator. On day 4, the medium was replaced with RPMI 1640 medium containing M-CSF, and on day 7, the mature macrophages were harvested.

### 2.6. Preparation of Apoptotic Cells

To generate apoptotic cells, Jurkat cells (purchased from ATCC, Manassas, USA) were cultured in RPMI 1640 medium without FCS, and apoptosis was induced with 0.5 *μ*g/ml staurosporine (BBI Life Science, Shanghai, China) for 3 h, as previously reported that the inhibitor of protein kinase staurosporine showed remarkable activity in inducing apoptosis in a wide variety of mammalian cells [23]. Afterwards, the cells were washed three times with PBS and resuspended in RPMI 1640 medium. Staurosporine treatment yielded a population of 90% apoptotic cells, which was verified by staining with annexin V and 7-amino-actinomycin D (7-AAD, Tianjin Sungene Biotech Co., China). Before being fed to the macrophages, the apoptotic cells were labeled with iFL Green dye (pHrodo, Invitrogen, Thermo Scientific, USA), which could be detected with FITC (fluorescein), protected from light at room temperature for 20 minutes.

### 2.7. Efferocytosis Assays

Human CD14-positive monocyte-derived macrophages were incubated with fresh medium containing 60 *μ*g/ml purified human Tyro3 antibody from SLE patients or unconjugated human IgG (BBI Life Science, Shanghai) for 1 h at 37°C in an incubator.

Then, the macrophages were incubated with staurosporine-induced apoptotic Jurkat cells labeled with iFL Green dye (ratioofmacrophages : apoptoticcells = 1 : 5) in RPMI 1640 medium without FCS in an incubator for 30 minutes. After being washed with PBS, the macrophages were collected using trypsin. Efferocytosis was determined according to the percentage of macrophages that phagocytosed apoptotic cells labeled with FITC using a FACS Canto II cytometer (BD Biosciences, San Jose, USA). The data were analyzed using FlowJo software (Tree Star Inc., Ashland, USA).

For immunofluorescence, the apoptotic cells were labeled with 5 *μ*M 5-(and 6)-carboxyfluorescein diacetate succinimidyl ester (CFSE) (eBioscience, Invitrogen, Thermo Scientific, USA), protected from light at room temperature for 10 minutes, and washed with PBS. Then, the macrophages were incubated with induced apoptotic Jurkat cells for 30 minutes. After washing with PBS, macrophages were fixed with 4% formaldehyde for 20 minutes. After three rinses with PBS, the cells were permeabilized with Triton X-100 (Beyotime, Shanghai, China) for 5 minutes. The cytoskeleton was dyed with phalloidin (Servicebio, Shanghai, China) and incubated for 1 h protected from light. DNA was stained with 10 *μ*g/ml 2-(4-amidinophenyl)-6-indolecarbamidine dihydrochloride (Hoechst 33324) (Thermo Scientific, USA) for 5 minutes protected from light. After rinsing 3 times with PBS, the cells were viewed by a confocal fluorescence microscope LSM 800 (ZEISS, Oberkochen, Germany).

### 2.8. Statistical Analysis

Continuous variables were tested for normality by the one-sample Kolmogorov-Smirnov test. Continuous variables were expressed as mean ± standarddeviation (SD) or median (interquartile range) as per distribution type, and categorical data were expressed as frequency and percentages. The receiver operating characteristic (ROC) curve and the area under the curve (AUC) were used to assess the sensitivity and specificity of anti-Tyro3 IgG for the diagnosis of SLE. Statistical analysis was performed using the independent samples *t*-test for normal data and the Mann-Whitney *U*-test for nonnormal data. The analyses were carried out under the two-sided principle. Correlations between groups were evaluated by Spearman correlation. A *p* value less than 0.05 was considered statistically significant. Graphs were drawn using GraphPad Prism (version 7, GraphPad Software Inc., San Diego, USA), and data were analyzed using the SPSS software for Windows (version 23; SPSS Inc., Chicago, USA).

## 3. Results

### 3.1. Increased Serum Levels of Tyro3 Receptor Autoantibody in Patients with SLE

**Figure 1:**
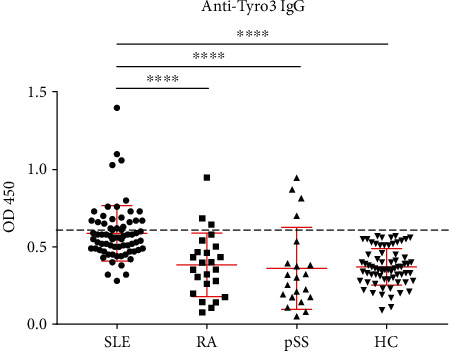
Detection of anti-Tyro3 IgG levels in SLE, RA, pSS, and HCs. SLE: systemic lupus erythematosus; RA: rheumatoid arthritis; pSS: primary Sjögren's Syndrome; HC: healthy control.

As TAM proteins are well-known kinase receptors that are crucial for macrophage efferocytosis for the clearance of apoptotic cells [10], to investigate the profile and clinical relevance of autoantibody against Tyro3 receptor in SLE, we detected the serum levels of anti-Tyro3 IgG in patients with SLE, RA, pSS, and HCs by ELISA. We recruited a total of 70 new-onset patients with SLE, and the clinical data are listed in Table 1. The results showed that the levels of anti-Tyro3 IgG were significantly higher in SLE patients than those in RA, pSS, and HCs (all *p* < 0.0001) (Figure 1). Then, we set a threshold using the mean control levels plus 2 SD for abnormal titers; the positive rate of anti-Tyro3 IgG in SLE, RA, and pSS was 24/70 (34.3%), 3/24 (12.5%), and 4/21 (19.0%), respectively, indicating that Tyro3 receptor antibody might be an important biomarker and play a role in the pathogenesis of SLE. Furthermore, the positivity of anti-Tyro3 in anti-Sm-negative patients was 16/53 (30.2%), while the positivity of which in anti-dsDNA-negative patients was 2/7 (28.6%). The results strengthen the significance of anti-Tyro3 antibody in the diagnosis of SLE. Besides, we detected serum from 11 patients before and after effective treatment by ELISA and there was no significant change of anti-Tyro3 antibody after effective treatment (*p* > 0.05, Supplementary Figure 1).

### 3.2. Association between Anti-Tyro3 IgG and the Clinical Manifestations in SLE Patients

**Figure 2:**
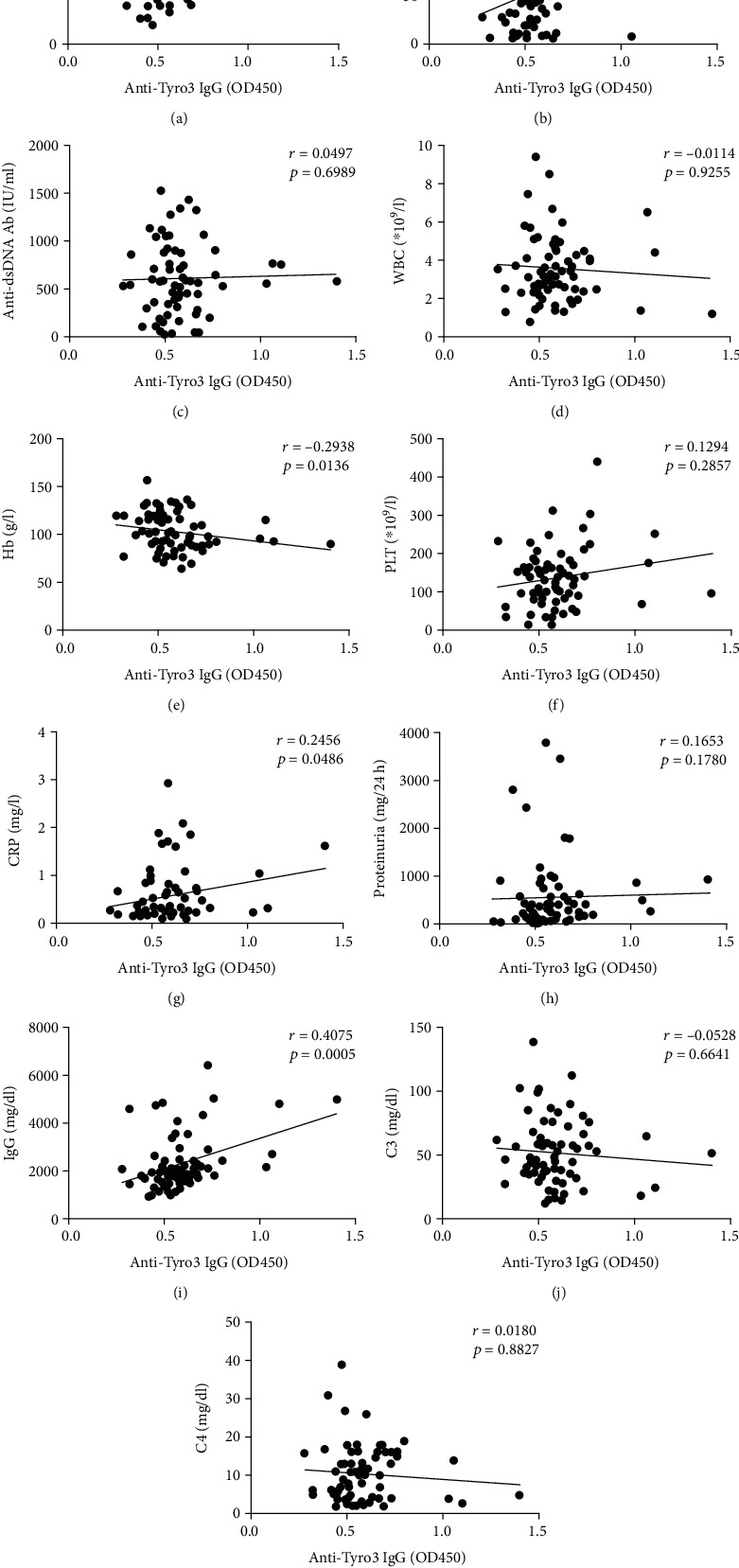
The correlation of anti-Tyro3 IgG levels and clinical manifestations in SLE patients. (a–k) The correlation between anti-Tyro3 IgG levels and SLEDAI score, ESR, Anti-dsDNA Ab, WBC, Hb, PLT, CRP, proteinuria, IgG, C3, and C4 in SLE patients. *p* < 0.05 represents a significant difference. SLE: systemic lupus erythematosus; HC: healthy control; ESR: erythrocyte sedimentation rate; SLEDAI: SLE disease activity index; anti-dsDNA Ab: anti-double-stranded DNA antibody; WBC: white blood cell; Hb: haemoglobin; PLT: platelet; CRP: C-reactive protein; IgG: immunoglobulin G; C3: complement 3; C4: complement 4.

Next, we analyzed the association of serum anti-Tyro3 IgG levels with the clinical data. The levels of anti-Tyro3 IgG were negatively associated with Hb (*r* = −0.294, *p* = 0.014) and positively correlated with the SLEDAI score (*r* = 0.254, *p* = 0.034), ESR (*r* = 0.430, *p* < 0.001), CRP (*r* = 0.246, *p* = 0.049), and IgG (*r* = 0.408, *p* = 0.001) (Figure 2). Furthermore, the differences of levels of anti-Tyro3 antibody in SLE patients with and without clinical characteristics were determined. As shown in Table 2, higher levels of anti-Tyro3 antibody were observed in patients with oral ulcers than patients without oral ulcers (*p* = 0.035).

We also detected autoantibodies against Axl and Mertk, which belong to TAM receptors, in the same cohort. The results showed that the levels of anti-Axl and anti-Mertk IgG were significantly higher in SLE patients than those in RA, pSS, and HCs (all *p* < 0.05, Supplementary Figures 2A and 2B). However, there was no correlation between anti-Axl IgG and SLEDAI score, neither did anti-Mertk IgG (all *p* > 0.05, Supplementary Figures 2C and 2D), indicating that the high levels of anti-Axl and anti-Mertk antibodies might not be associated with SLE disease activity.

### 3.3. Receiver Operating Characteristic Curves of Anti-Tyro3 IgG

**Figure 3:**
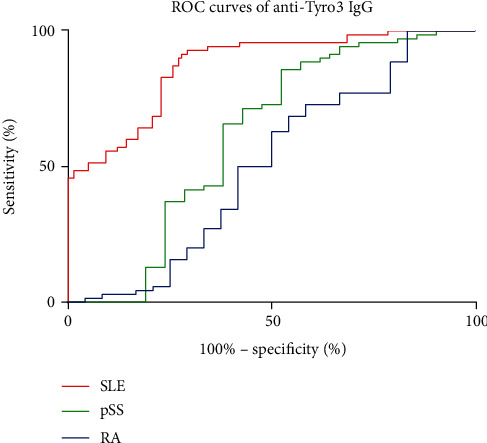
The receiver operating characteristic (ROC) curves of anti-Tyro3 IgG in SLE, RA, pSS, and HCs. The areas under the curve (AUC) of anti-Tyro3 IgG in SLE, RA, and pSS were 0.8708 (95% CI: 0.8136-0.9281) (*p* < 0.0001), 0.5048 (95% CI: 0.3512-0.6583) (*p* = 0.9447), and 0.6146 (95% CI: 0.4468-0.7824) (*p* = 0.1125), respectively. SLE: systemic lupus erythematosus; RA: rheumatoid arthritis; pSS: primary Sjögren's Syndrome.

To determine the efficacy of the measurement of autoantibody against Tyro3 receptor for diagnosing SLE, we calculated the ROC curves to determine the sensitivity and specificity of the autoantibody in distinguishing patients with SLE, RA, and pSS from HCs. As shown in Figure 3, the AUCs of anti-Tyro3 IgG in SLE, RA, and pSS were 0.8708 (95% CI: 0.8136-0.9281) (*p* < 0.0001), 0.5048 (95% CI: 0.3512-0.6583) (*p* = 0.9447), and 0.6146 (95% CI: 0.4468-0.7824) (*p* = 0.1125), respectively. ROC curves illustrated that the anti-Tyro3 antibody could differentiate patients with SLE from HCs.

### 3.4. SLE Patient-Derived Autoantibody against Tyro3 Receptor Inhibited the Efferocytosis of Macrophages

**Figure 4:**
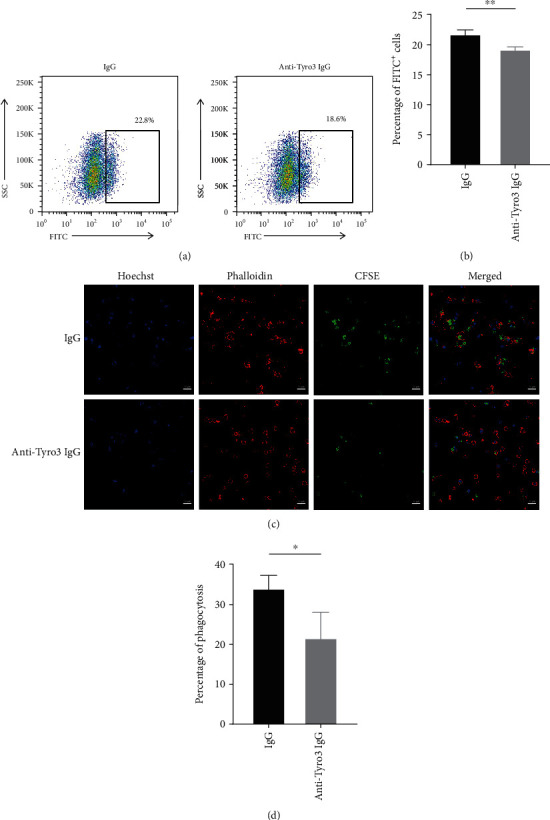
Autoantibody against Tyro3 receptor reduced macrophage efferocytosis by flow cytometry and immunofluorescence. (a) The efferocytosis of macrophages was analyzed by flow cytometry. (b) The statistical graph showing the flow cytometry data (*n* = 4). The values represent the mean ± SD. ^∗∗^*p* < 0.01. (c) Representative photograph of the efferocytosis of macrophages monitored by immunofluorescence, presented as merged pictures of Hoechst (blue), phalloidin (red), and CFSE (green). Bar, 20 *μ*m. (d) Statistical data of the percentage of efferocytosis in 100x views of the confocal microscope (*n* = 3). The values represent the mean ± SD. ^∗^*p* < 0.05.

Since TAM receptors mainly functioned in macrophage-associated efferocytosis, we further determined the role of Tyro3 receptor autoantibody in the efferocytosis of macrophages. First, we purified specific IgG-type antibody against Tyro3 from the serum of new-onset SLE patients with high optical density (OD) values at 450 nm, which was further confirmed by silver staining compared to different quantity of unconjugated human IgG (Supplementary Figure 3). Furthermore, we explored the cross-reaction between purified anti-Tyro3 antibody and TAM receptors and CD14, a surface antigen preferentially expressed mainly on monocytes/macrophages, by ELISA assay following the standard procedure (Supplementary Table 1). Immunoprecipitation was also used to verify the binding of purified anti-Tyro3 IgG to recombinant human Tyro3 protein (Supplementary Figure 4). Then, primary human monocyte-derived macrophages and staurosporine-induced apoptotic Jurkat cells were incubated with purified anti-Tyro3 IgG or unconjugated human IgG, and macrophage efferocytosis was analyzed by flow cytometry and immunofluorescence. As shown in Figure 4,macrophage efferocytosis was significantly decreased after anti-Tyro3 IgG (*p* = 0.004) treatment compared with unconjugated human IgG treatment detected by flow cytometry. Furthermore, immunofluorescence assay showed a decreased engulfment of apoptotic cells in the macrophages incubated with purified anti-Tyro3 IgG (*p* = 0.044) compared with unconjugated human IgG. These data suggested that Tyro3 receptor autoantibody reduced the efferocytosis of macrophages by blocking Tyro3 receptor and might result in the accumulation of cell debris, thus might be involved in the pathogenesis of SLE.

## 4. Discussion

The overproduction of autoantibodies is a hallmark of the pathogenesis of SLE. The contribution of the dysregulation of macrophages to autoantibody overproduction in SLE has been widely studied, but the effect of autoantibodies on macrophages in SLE has rarely been reported. It was reported that these antibodies could affect many cells via direct binding to antigens on cells [24]. The functions of these autoantibodies included the activation or inhibition of downstream signaling and the blocking of interactions between targets and other proteins [25]. Recently, our team found that the overproduced antibodies in SLE included antibodies recognizing proteins specifically expressed in immune cells that were responsible for key immune responses, such as anti-PD-1 antibody [26]. These immune response-related antibodies might further disturb the balance of the immune system and contribute to the progression of SLE. Here, we demonstrated that patients with SLE produced high levels of autoantibody against Tyro3, which was one of the three key tyrosine kinases involved in the macrophage-mediated elimination of apoptotic cells. Besides, anti-Tyro3 antibody was associated with the SLEDAI score, ESR, and CRP, indicating that the antibody was related to SLE disease activity. Further functional studies revealed that anti-Tyro3 IgG inhibited the efferocytosis of macrophages, which might increase the accumulation of autoantigen and further promote the production of autoantibodies in SLE.

The major factor involved in the regulation of macrophage-mediated efferocytosis is TAM receptors [11, 13, 27]. Our study also detected the levels of anti-Axl and anti-Mertk IgG, which were significantly higher in SLE patients than those in RA, pSS, and HCs. However, there was no correlation between anti-Axl IgG and SLEDAI score, neither did anti-Mertk IgG. It indicated that only the levels of anti-Tyro3 IgG were associated with disease activity of SLE, supporting it as a potential novel disease biomarker.

It was interesting that the statistical analysis showed that patients with the presence of oral ulcers had higher levels of anti-Tyro3 IgG, and there was little known about the relationship between TAM autoantibodies and oral ulcers. It has been reported that CD68-positive macrophages are one of the main inflammatory cells relevant to the pathogenesis of ulcers [28]. In oral ulcers, the apoptosis of epithelium remains in large quantity and poses a great pressure of scavenging cells such as macrophages. The rapidly apoptotic epithelial cell may exceed the ability of phagocytosis of macrophages, which lead to sloughing of the dead epithelial cells [29]. Furthermore, autoantibody-related defective phagocytosis of macrophages might in turn worsen the symptom of oral ulcers.

The identification of pathological antibodies is key to understand the abnormal events that occur in patients with SLE. Our study is the first to report the enrichment of pathological anti-Tyro3 antibody in SLE and its association with disease activity. We also indicated that autoantibody against Tyro3 might be responsible for the reduced efferocytosis of macrophages.

## 5. Conclusions

This study showed an elevated level of anti-Tyro3 IgG in patients with SLE compared to HCs and was associated with SLE disease activity, indicating anti-Tyro3 antibody a novel disease biomarker. In addition, our results demonstrated that autoantibody against Tyro3 impaired efferocytosis of macrophages, which might be involved in the pathogenesis of SLE.

## Figures and Tables

**Table 1:** The clinical and laboratory characteristics of new-onset and treatment-naïve SLE patients.

Variable	SLE patients
Gender (female/male, *n*)	60/10
Age (mean ± SD, years)	39 ± 16
Disease duration (months, median, IQR)	3.5 (1, 24)
SLEDAI (mean ± SD)	12 ± 5
Fever (*n*, %)	32 (45.7)
Arthritis (*n*, %)	42 (60.0)
Rash (*n*, %)	36 (51.4)
Oral ulcer (*n*, %)	16 (22.9)
Alopecia (*n*, %)	16 (22.9)
Vasculitis (*n*, %)	5 (7.1)
Serositis (*n*, %)	16 (22.9)
Photosensitivity (*n*, %)	10 (14.3)
Raynaud's phenomenon (*n*, %)	13 (18.6)
Hematological (*n*, %)	54 (77.1)
Lupus nephritis (proteinuria ≥ 0.5 g/24 h) (*n*, %)	31 (44.3)
Neuropsychiatric manifestations (*n*, %)	1 (1.4)
Anti-dsDNA positive (*n*, %)	63 (90.0)
Anti-Sm positive (*n*, %)	17 (24.3)
Anti-SSA positive (*n*, %)	38 (54.3)
Anti-SSB positive (*n*, %)	8 (11.4)
Anti-U1RNP positive (*n*, %)	27 (38.6)
Anti-Rib-P positive (*n*, %)	29 (41.4)
Anti-nucleosome-A positive (*n*, %)	24 (34.3)

Note: SD: standard deviation; SLE: systemic lupus erythematosus; SLEDAI: SLE disease activity index; IQR: interquartile range; dsDNA: double-stranded DNA.

**Table 2:** Comparison of anti-Tyro3 antibody according to disease manifestations in 70 new-onset and treatment-naïve SLE patients.

	Anti-Tyro3 IgG (OD 450)		*p* value
Photosensitivity	(+), *n* = 10	0.645 ± 0.185	0.185
	(-), *n* = 60	0.578 ± 0.177	
Raynaud's phenomenon	(+), *n* = 13	0.594 ± 0.187	0.898
	(-), *n* = 57	0.586 ± 0.178	
Fever	(+), *n* = 32	0.618 ± 0.228	0.402
	(-), *n* = 38	0.562 ± 0.121	
Serositis	(+), *n* = 16	0.606 ± 0.153	0.243
	(-), *n* = 54	0.582 ± 0.187	
Oral ulcer	(+), *n* = 16	0.698 ± 0.264	0.035^∗^
	(-), *n* = 54	0.555 ± 0.131	
Rash	(+), *n* = 36	0.605 ± 0.214	0.953
	(-), *n* = 34	0.569 ± 0.132	
Alopecia	(+), *n* = 16	0.599 ± 0.157	0.796
	(-), *n* = 54	0.584 ± 0.186	
Arthritis	(+), *n* = 42	0.607 ± 0.178	0.148
	(-), *n* = 28	0.558 ± 0.179	
Vasculitis	(+), *n* = 5	0.634 ± 0.166	0.367
	(-), *n* = 65	0.584 ± 0.180	

Note: anti-Tyro3 IgG (OD 450) are shown as mean ± SD, and differences between two groups were analyzed using the independent samples *t*-test for normal data and the Mann-Whitney *U* test for nonnormal data. SLE: systemic lupus erythematosus. ^∗^*p* < 0.05.

## Data Availability

The datasets generated and/or analyzed during the current study are available from the corresponding authors on reasonable request.

## Supplementary Materials

Supplementary Materials Supplementary Table 1: cross-reaction between purified anti-Tyro3 antibody and TAM receptors and CD14 by ELISA. Supplementary Figure 1: the levels of anti-Tyro3 IgG in SLE before and after effective treatment. Supplementary Figure 2: the levels of anti-Axl and anti-Mertk IgG in patients with SLE, RA, pSS, and HCs and its association with the SLEDAI score. (A) The levels of anti-Axl IgG in patients with SLE, RA, pSS, and HCs detected by ELISA assay; (B) the levels of anti-Mertk IgG in patients with SLE, RA, pSS, and HCs detected by ELISA assay; (C) the correlation between anti-Axl IgG and SLEDAI score; (D) the correlation between anti-Mertk IgG and SLEDAI score. SLE: systemic lupus erythematosus; RA: rheumatoid arthritis; pSS: primary Sjögren's Syndrome; HC: healthy control; SLEDAI: SLE disease activity index. ^∗^*p* < 0.05, ^∗∗^*p* < 0.01, and ^∗∗∗∗^*p* < 0.0001. Supplementary Figure 3: silver staining of purified anti-Tyro3 IgG from SLE patients. The heavy chain and light chain of IgG were 50 kD and 25 kD, respectively. The quantity of IgG-1, IgG-2, and IgG-3 was 1 *μ*g, 0.3 *μ*g, and 0.1 *μ*g, respectively. The quantity of anti-Tyro3 IgG was 0.3 *μ*g. IgG: unconjugated human IgG. Supplementary Figure 4: immunoprecipitation of recombinant human Tyro3 protein and purified anti-Tyro3 IgG vs. unconjugated human IgG using Protein A/G PLUS-Agarose. Tyro3: recombinant human Tyro3 protein; human IgG: unconjugated human IgG.

## References

[1] Tsokos G. C., Lo M. S., Reis P. C., Sullivan K. E. (2016). New insights into the immunopathogenesis of systemic lupus erythematosus. *Nature Reviews Rheumatology*.

[2] Kaul A., Gordon C., Crow M. K. (2016). Systemic lupus erythematosus. *Nature Reviews Disease Primers*.

[3] Li Y., Lee P. Y., Reeves W. H. (2010). Monocyte and macrophage abnormalities in systemic lupus erythematosus. *Archivum Immunologiae et Therapiae Experimentalis*.

[4] Muñoz L. E., Lauber K., Schiller M., Manfredi A. A., Herrmann M. (2010). The role of defective clearance of apoptotic cells in systemic autoimmunity. *Nature Reviews Rheumatology*.

[5] Poon I. K. H., Lucas C. D., Rossi A. G., Ravichandran K. S. (2014). Apoptotic cell clearance: basic biology and therapeutic potential. *Nature Reviews Immunology*.

[6] Chen X., Shen Y., Sun C., Wu F. X., Chen Y., Yang C. D. (2011). Anti-class a scavenger receptor autoantibodies from systemic lupus erythematosus patients impair phagocytic clearance of apoptotic cells by macrophages in vitro. *Arthritis research & therapy*.

[7] Barrera-Vargas A., Gómez-Martín D., Carmona-Rivera C. (2018). Differential ubiquitination in NETs regulates macrophage responses in systemic lupus erythematosus. *Annals of the Rheumatic Diseases*.

[8] Blander J. M. (2017). The many ways tissue phagocytes respond to dying cells. *Immunological Reviews*.

[9] Luo B., Gan W., Liu Z. (2016). Erythropoeitin signaling in macrophages promotes dying cell clearance and immune tolerance. *Immunity*.

[10] Graham D. K., DeRyckere D., Davies K. D., Earp H. S. (2014). The TAM family: phosphatidylserine sensing receptor tyrosine kinases gone awry in cancer. *Nature Reviews Cancer*.

[11] Zagórska A., Través P. G., Lew E. D., Dransfield I., Lemke G. (2014). Diversification of TAM receptor tyrosine kinase function. *Nature Immunology*.

[12] van der Meer J. H. M., van der Poll T., van ‘t Veer C. (2014). TAM receptors, Gas6, and protein S: roles in inflammation and hemostasis. *Blood*.

[13] Rothlin C. V., Lemke G. (2010). TAM receptor signaling and autoimmune disease. *Current Opinion in Immunology*.

[14] Cohen P. L., Caricchio R., Abraham V. (2002). Delayed apoptotic cell clearance and lupus-like autoimmunity in mice lacking the c-mer membrane tyrosine kinase. *Journal of Experimental Medicine*.

[15] Rothlin C. V., Carrera-Silva E. A., Bosurgi L., Ghosh S. (2015). TAM receptor signaling in immune homeostasis. *Annual Review of Immunology*.

[16] Lu Q., Lemke G. (2001). Homeostatic regulation of the immune system by receptor tyrosine kinases of the tyro 3 family. *Science*.

[17] Smart S., Vasileiadi E., Wang X., DeRyckere D., Graham D. (2018). The emerging role of TYRO3 as a therapeutic target in cancer. *Cancers*.

[18] Wu J., Ekman C., Jönsen A. (2011). Increased plasma levels of the soluble Mer tyrosine kinase receptor in systemic lupus erythematosus relate to disease activity and nephritis. *Arthritis Research & Therapy*.

[19] Recarte-Pelz P., Tàssies D., Espinosa G. (2013). Vitamin K-dependent proteins GAS6 and protein S and TAM receptors in patients of systemic lupus erythematosus: correlation with common genetic variants and disease activity. *Arthritis Research & Therapy*.

[20] Ballantine L., Midgley A., Harris D., Richards E., Burgess S., Beresford M. W. (2015). Increased soluble phagocytic receptors sMer, sTyro3 and sAxl and reduced phagocytosis in juvenile-onset systemic lupus erythematosus. *Pediatric Rheumatology*.

[21] Hochberg M. C. (1997). Updating the American College of Rheumatology revised criteria for the classification of systemic lupus erythematosus. *Arthritis & Rheumatology*.

[22] Gladman D. D., Ibañez D., Urowitz M. B. (2002). Systemic lupus erythematosus disease activity index 2000. *Journal of Rheumatology*.

[23] Cusinato F., Pighin I., Luciani S., Trevisi L. (2006). Synergism between staurosporine and drugs inducing endoplasmic reticulum stress. *Biochemical Pharmacology*.

[24] Racanelli V., Prete M., Musaraj G., Dammacco F., Perosa F. (2011). Autoantibodies to intracellular antigens: generation and pathogenetic role. *Autoimmunity Reviews*.

[25] Nimmerjahn F., Ravetch J. V. (2010). Antibody-mediated modulation of immune responses. *Immunological Reviews*.

[26] Shi H., Ye J., Teng J. (2017). Elevated serum autoantibodies against co-inhibitory PD-1 facilitate T cell proliferation and correlate with disease activity in new-onset systemic lupus erythematosus patients. *Arthritis Research & Therapy*.

[27] Fourgeaud L., Través P. G., Tufail Y. (2016). TAM receptors regulate multiple features of microglial physiology. *Nature*.

[28] Delgado W. A., Almeida O. P., Vargas P. A., León J. E. (2009). Oral ulcers in HIV-positive Peruvian patients: an immunohistochemical and in situ hybridization study. *Journal of Oral Pathology & Medicine*.

[29] Al-Samadi A., Drozd A., Salem A., Hietanen J., Häyrinen-Immonen R., Konttinen Y. T. (2015). Epithelial cell apoptosis in recurrent aphthous ulcers. *Journal of Dental Research*.

