# Supplementary material for: Anti-Tyro3 IgG Associates with Disease Activity and Reduces Efferocytosis of Macrophages in New-Onset Systemic Lupus Erythematosus
Source: J Immunol Res. 2020 Nov 10;2020:2180708. doi: 10.1155/2020/2180708 (PMC7673931; doi:10.1155/2020/2180708)
Supplement: Supplementary Materials — Supplementary Table 1: cross-reaction between purified anti-Tyro3 antibody and TAM receptors and CD14 by ELISA. Supplementary Figure 1: the levels of anti-Tyro3 IgG in SLE before and after effective treatment. Supplementary Figure 2: the levels of anti-Axl and anti-Mertk IgG in patients with SLE, RA, pSS, and HCs and its association with the SLEDAI score. (A) The levels of anti-Axl IgG in patients with SLE, RA, pSS, and HCs detected by ELISA assay; (B) the levels of anti-Mertk IgG in patients with SLE, RA, pSS, and HCs detected by ELISA assay; (C) the correlation between anti-Axl IgG and SLEDAI score; (D) the correlation between anti-Mertk IgG and SLEDAI score. SLE: systemic lupus erythematosus; RA: rheumatoid arthritis; pSS: primary Sjögren's Syndrome; HC: healthy control; SLEDAI: SLE disease activity index. ∗p < 0.05, ∗∗p < 0.01, and ∗∗∗∗p < 0.0001. Supplementary Figure 3: silver staining of purified anti-Tyro3 IgG from SLE patients. The heavy chain and light chain of IgG were 50 kD and 25 kD, respectively. The quantity of IgG-1, IgG-2, and IgG-3 was 1 μg, 0.3 μg, and 0.1 μg, respectively. The quantity of anti-Tyro3 IgG was 0.3 μg. IgG: unconjugated human IgG. Supplementary Figure 4: immunoprecipitation of recombinant human Tyro3 protein and purified anti-Tyro3 IgG vs. unconjugated human IgG using Protein A/G PLUS-Agarose. Tyro3: recombinant human Tyro3 protein; human IgG: unconjugated human IgG. [file 2180708.f1.doc]

**Supplementary table 1.** Cross-reaction between purified anti-Tyro3 antibody and TAM receptors and CD14 by ELISA.

| OD (450nm) | Anti-Tyro3 antibody |
| --- | --- |
| recombinant human Tyro3 protein | 0.749 |
| recombinant human Axl protein | 0.075 |
| recombinant human Mertk protein | 0.077 |
| recombinant human CD14 protein | 0.071 |


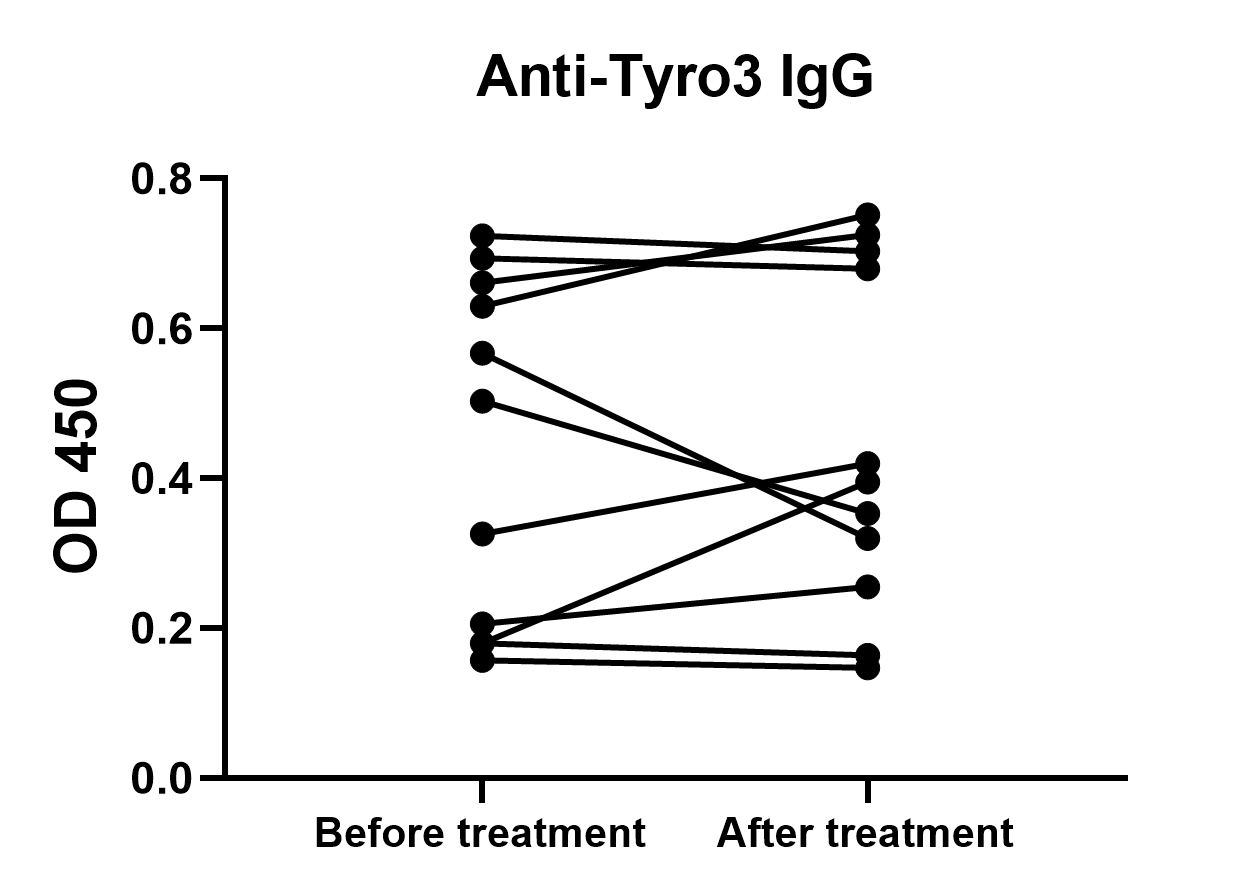


**Supplementary figure 1.** The levels of anti-Tyro3 IgG in SLE before and after effective treatment.


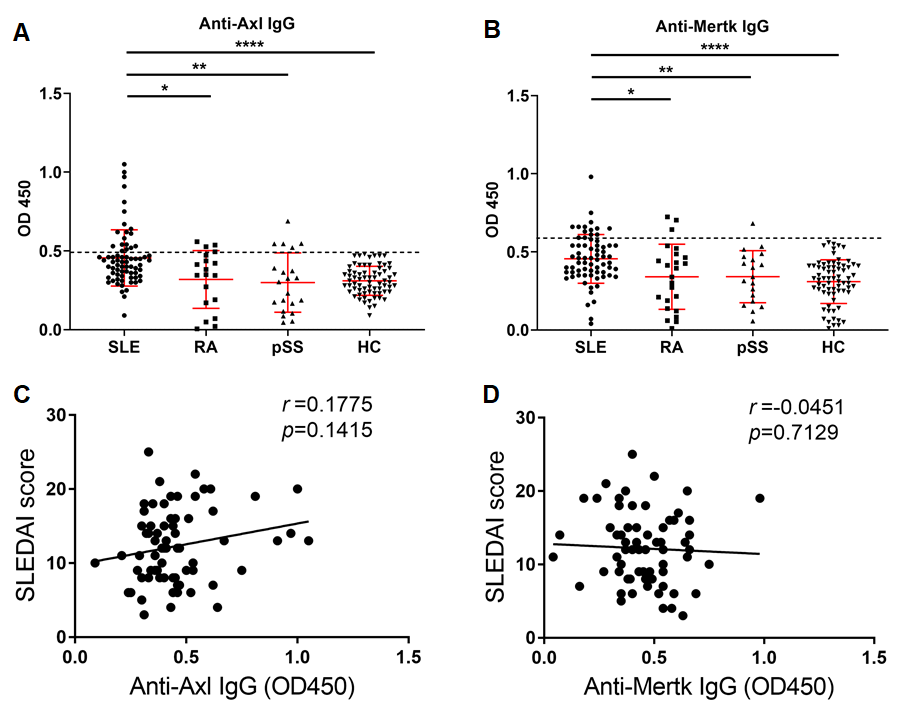


**Supplementary figure 2.** The levels of anti-Axl and anti-Mertk IgG in patients with SLE, RA, pSS and HCs and its association with SLEDAI score. (A) The levels of anti-Axl IgG in patients with SLE, RA, pSS and HCs detected by ELISA assay; (B) The levels of anti-Mertk IgG in patients with SLE, RA, pSS and HCs detected by ELISA assay; (C) The correlation between anti-Axl IgG and SLEDAI score; (D) The correlation between anti-Mertk IgG and SLEDAI score. SLE, systemic lupus erythematosus; RA, rheumatoid arthritis; pSS, primary Sjögren’s Syndrome; HC, healthy control; SLEDAI, SLE disease activity index. * *p* < 0.05, ** *p* < 0.01, **** *p* < 0.0001.


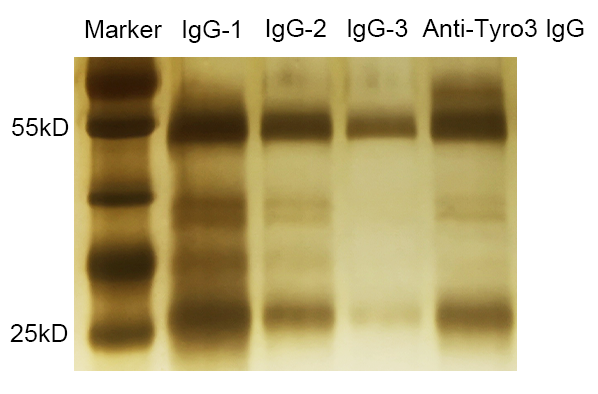


**Supplementary figure 3.** Silver Staining of purified anti-Tyro3 IgG from SLE patients. The heavy chain and light chain of IgG were 50 kD and 25 kD, respectively. The quantity of IgG-1, IgG-2, IgG-3 were 1 μg, 0.3 μg, 0.1 μg, respectively. The quantity of anti-Tyro3 IgG was 0.3 μg. IgG, unconjugated human IgG.


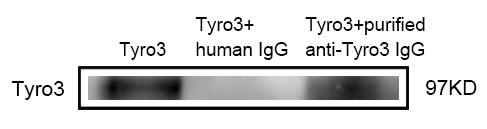


**Supplementary figure 4.** Immunoprecipitation of recombinant human Tyro3 protein and purified anti-Tyro3 IgG vs unconjugated human IgG using Protein A/G PLUS-Agarose. Tyro3, recombinant human Tyro3 protein; human IgG, unconjugated human IgG.
